# Loss of Caveolin-1 Impairs Light Flicker-Induced Neurovascular Coupling at the Optic Nerve Head

**DOI:** 10.3389/fnins.2021.764898

**Published:** 2021-11-08

**Authors:** Jing Hong Loo, Ying Shi Lee, Chang Yi Woon, Victor H. K. Yong, Bingyao Tan, Leopold Schmetterer, Rachel S. Chong

**Affiliations:** ^1^Yong Loo Lin School of Medicine, National University of Singapore, Singapore, Singapore; ^2^Singapore Eye Research Institute, Singapore, Singapore; ^3^School of Chemical and Biomedical Engineering, Nanyang Technological University, Singapore, Singapore; ^4^SERI-NTU Advanced Ocular Engineering (STANCE), Singapore, Singapore; ^5^Institute of Molecular and Clinical Ophthalmology Basel, Basel, Switzerland; ^6^Department of Clinical Pharmacology, Center for Medical Physics and Biomedical Engineering, Medical University of Vienna, Vienna, Austria; ^7^Duke-National University of Singapore Graduate Medical School, Singapore, Singapore

**Keywords:** glaucoma, neurovascular coupling, caveolin, neuroprotection, neurovascular dysfunction

## Abstract

Glaucoma is a neurodegenerative disease, which results in characteristic visual field defects. Intraocular pressure (IOP) remains the main risk factor for this leading cause of blindness. Recent studies suggest that disturbances in neurovascular coupling (NVC) may be associated with glaucoma. The resultant imbalance between vascular perfusion and neuronal stimulation in the eye may precede retinal ganglion cell (RGC) loss and increase the susceptibility of the eye to raised IOP and glaucomatous degeneration. Caveolin-1 (Cav-1) is an integral scaffolding membrane protein found abundantly in retinal glial and vascular tissues, with possible involvement in regulating the neurovascular coupling response. Mutations in Cav-1 have been identified as a major genetic risk factor for glaucoma. Therefore, we aim to evaluate the effects of Cav-1 depletion on neurovascular coupling, retinal vessel characteristics, RGC density and the positive scotopic threshold response (pSTR) in Cav-1 knockout (KO) versus wild type C57/Bl6 mice (WT). Following light flicker stimulation of the retina, Cav-1 KO mice showed a smaller increase in perfusion at the optic nerve head and peripapillary arteries, suggesting defective neurovascular coupling. Evaluation of the superficial capillary plexus in Cav-1 KO mice also revealed significant differences in vascular morphology with higher vessel density, junction density and decreased average vessel length. Cav-1 KO mice exhibited higher IOP and lower pSTR amplitude. However, there was no significant difference in RGC density between Cav-1 KO and wild type mice. These findings highlight the role of Cav-1 in regulating neurovascular coupling and IOP and suggest that the loss of Cav-1 may predispose to vascular dysfunction and decreased RGC signaling in the absence of structural loss. Current treatment for glaucoma relies heavily on IOP-lowering drugs, however, there is an immense potential for new therapeutic strategies that increase Cav-1 expression or augment its downstream signaling in order to avert vascular dysfunction and glaucomatous change.

## Introduction

Glaucoma is the second leading cause of irreversible blindness worldwide (GBD 2019 Blindness and Vision Impairment Collaborators, and Vision Loss Expert Group of the Global Burden of Disease Study, 2021). The global prevalence of glaucoma stands at 3.54%, with an estimated 76.0 million people suffering from it (Tham et al., 2014), resulting in a significant public health and economic burden (Varma et al., 2011). Primary open angle glaucoma, which is the most common form of glaucoma, represents a diagnostic and treatment challenge because it progresses insidiously and leads to characteristic retinal ganglion cell (RGC) loss and visual field defects only later on in the course of the disease (Cohen and Pasquale, 2014). For decades, raised intraocular pressure (IOP) has been recognized as the major modifiable risk factor of open angle glaucoma (Nemesure et al., 2007). However, a significant proportion of glaucoma patients do not have elevated IOP (Prum et al., 2016), nor does elevated IOP necessarily lead to glaucomatous degeneration (Heijl et al., 2002). Therefore, it appears that other mechanisms might be at play in the pathogenesis of glaucoma.

Early studies have suggested the possibility of vascular dysregulation as an IOP-independent factor in the pathogenesis of glaucoma (Flammer et al., 2002). Changes in microvascular structural characteristics, especially the narrowing of vessel caliber and loss of peripapillary and macula vessel density, have been observed in epidemiological studies (Wan et al., 2018; Rolle et al., 2019). However, the temporal association between vascular dysfunction and optic neuropathy remains unclear. Numerous theories have been proposed to explain the observed vascular dysregulation in glaucoma. Chronic arterial hypertension may cause vascular sclerosis, compromising vasodilation or constriction as part of autoregulatory functions; alternatively, micro-ischemic events may contribute to arterial vasospasm and fluctuations in perfusion (Anderson, 1999).

To date, the structural changes in retina microvasculature and RGC have been well-described, but less is known of the functional vascular changes precipitating glaucomatous progression. Emerging evidence suggests that diminished electrical activity of RGC occurs before measurable retinal nerve fiber loss (Porciatti and Ventura, 2012), although the association between glaucoma and microvascular function in the eye remains largely unknown. There exists an intricate interplay between vascular cells, macroglia and retinal ganglion cells, collectively termed the neurovascular unit of the eye, that allows for vascular autoregulation via neurovascular coupling (NVC) (Wareham and Calkins, 2020). In healthy retinal tissue, NVC enables blood flow to be tightly matched with metabolic requirements via vasodilatory mechanisms within retinal vascular beds. Previous clinical studies have described defective NVC in primary open-angle glaucoma leading to an imbalance between ocular perfusion and neuronal stimulation (Garhöfer et al., 2004; Riva et al., 2004).

Mutations in the caveolin-1 (Cav-1) gene have been found to be associated with an increase genetic risk of open-angle glaucoma (Wiggs et al., 2011). This can be attributed to its involvement in raised IOP, with possible mechanisms such as overactive nitric oxide synthase signaling pathways (De Ieso et al., 2020) and decreased aqueous humor drainage in the anterior chamber (Elliott et al., 2016). Additionally, Cav-1 is found abundantly amongst retinal vascular and glial cells that mediate the aforementioned NVC response (Gu et al., 2014b). Hence, it is possible that Cav-1 might contribute to glaucomatous degeneration through defective NVC on top of raised IOP.

In the current study we aim to evaluate the effects of Cav-1 depletion on neurovascular coupling at the optic nerve head, retinal vessel morphology, RGC density and function.

## Materials and Methods

### Animals

All animals were treated in accordance with the ARVO Statement for the Use of Animals in Ophthalmic and Vision Research. This study was approved by the SingHealth Institutional Animal Care and Use Committee (SHS/2017/SHS/1354). Adult male C57/Bl6 mice (WT, *n* = 10, C57BL/6J from InVivos) and Caveolin-1 knockout mice (Cav-1 KO, *n* = 10, #007083 from JAX Lab) were used. Experimentation commenced at 12 weeks of age. Mice were anesthetized by intraperitoneal injections of 0.1 mL ketamine/xylazine mixture comprising 2 mg/mL xylazine (Troy Laboratories Pte Ltd., Glendenning, NSW, Australia) and 20 mg/mL ketamine (Ceva Animal Health Pty Ltd., Glenorie, NSW, Australia) for intraocular pressure measurements, imaging and electroretinogram testing.

### Intraocular Pressure Measurements and Optic Nerve Head Photography

An Icare TONOLAB rebound tonometer (Icare Finland Oy, Vantaa, Finland) was used to measure IOP (*n* = 10, each) between 8 AM and 10 AM, with the room lighting at approximately 700 lux; an average of 5 measurements were taken each time per eye. Optic nerve head imaging was performed following pupil dilation with 1% tropicamide (Mydriacyl, Alcon Laboratories, Inc., Fort Worth, TX, United States). Digital color photographs of the optic nerve head were taken using the Micron IV retinal imaging microscope (Phoenix Research Laboratories, Pleasanton, CA, United States).

### Laser Speckle Flowgraphy

Retinal blood flow around the ONH region in the both eyes (from *n* = 10 mice, each) was measured with laser speckle flowgraphy (LSFG) (LSFG-Micro system, Softcare Co., Ltd., Fukutsu, Japan). This system comprises a charge-coupled device (CCD) camera equipped with a diode laser that is attached to a microscope (SZ61, Olympus Corporation, Tokyo, Japan). Eyes were dark adapted for 2 h before exposure to light flicker conditions following dilation with 1% tropicamide (Mydriacyl, Alcon Laboratories, Inc., Fort Worth, TX, United States) using a white LED light, set at 30 Lux, 12 Hz and for 3 min based on previous studies demonstrating maximum increases in retinal blood flow under these conditions (Hanaguri et al., 2020). Body temperature was maintained at 37°C using a heat pad placed on top of the microscope stage. The mean blur rate (MBR) images, which represents a relative index of blood velocity, were acquired continuously at a rate of 30 frames/sec for 4 s. Three consecutive measurements were performed without altering the position of the mice. MBR were measured from large blood vessels and microvascular neuro-retinal tissues at the optic nerve head, as well as from the peripapillary arteries and veins, calculated with LSFG analyzer software (version 3.1.58.0, Softcare Co., Ltd., Fukutsu, Japan). Arteries and veins were determined based on their position and appearance in the color photographs, where arteries were narrower, lighter colored and more reflectant while the converse was true for veins. The MBR outcomes were expressed in terms of mean blood flow over three measurements in arbitrary units (AU), as well as percentage change in blood flow following 3 min of exposure to flicker stimulus from the baseline. Vessel density of the optic nerve head determined by the LSFG analyzer software was also expressed as a percentage of the total area.

### Electroretinography

Mice (*n* = 10, each) were dark adapted for a minimum of 12 h. All electroretinography (ERG) recordings were performed using the Espion Visual Electrophysiology System (Diagnosys LLC, Westford, MA, United States) in a dark room under dim red light illumination. Mice were anaesthetized and their pupils dilated as above. Body temperature was maintained at 37°C using a heat pad fixed on the ERG stage. Vidisic coupling gel (Bausch & Lomb Pharmaceuticals, Inc., Tampa, FL, United States) was applied to each eye, and a customized platinum wire electrode (#V0352 from ProSciTech Pty Ltd., Queensland, Australia) was placed against the corneal surface of both eyes to record electrical signals. A customized reference electrode (#V0352 from ProSciTech Pty Ltd., Queensland, Australia) placed around both eyes, near to limbus, and a stainless steel subdermal needle electrodes (Xenotec Inc., DBA OcuScience, Henderson, NV, United States) was inserted into the tail as the ground electrode. The mouse head was positioned in front of a Ganzfeld stimulus, and the responses were recorded as follows. Positive scotopic threshold response (pSTR) was defined as the peak amplitude of waveforms recorded at stimulus intensities of −6.0, −5.45, −5.30 log cd.s/m^2^. At each intensity, 25 flashes with an inter-stimulus interval of 3,000 ms were averaged. The mean peak amplitude of each waveform across the different stimulus intensities was calculated.

### Immunohistochemistry for Retinal Ganglion Cell Density and Vessel Morphology

Mouse eyes were enucleated and processed for retinal flatmounts or cryosections. Retinas were harvested for flatmounts (*n* = 10 each, C57 or Cav-1KO) and fixed in 4% paraformaldehyde before staining for the retinal ganglion cell (RGC) marker RBPMS (RNA-binding protein with multiple splicing, GTX1186119; GeneTex Inc., Irvine, CA, United States) and endothelial cell marker Isolectin-B4 (#132450, Invitrogen, Thermo Fisher Scientific, Waltham, MA, United States). Images were taken at three locations of increasing eccentricity (central ∼100 μm, mid ∼735 μm, peripheral ∼1370 μm) from the optic nerve head in each quadrant at 20× using an Olympus FV3000 confocal microscope (Olympus Corporation, Tokyo, Japan). To determine RGC density, RBPMS-positive nuclei in each image were counted by a masked observer and divided by the area of retina covered in mm2. Flatmount images of Isolectin-B4 staining were assessed using Angiotool to determine the vessel density, junction density in terms of the percentage over total retina area, and average vessel length in μm (Zudaire et al., 2011).

### Statistical Analysis

Unpaired *t*-tests were conducted using Prism 9 software (GraphPad Software, San Diego, CA, United States). Group data are given as mean ± SEM, where statistical significance was assumed by *p* < 0.05^∗^, *p* < 0.01^∗∗^, *p* < 0.001^∗∗∗^, *p* < 0.0001^****^.

## Results

### Intraocular Pressure Measurements

**FIGURE 1:**
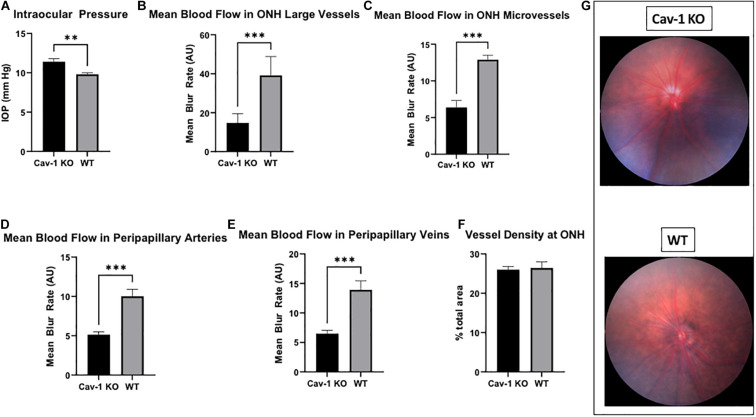
Intraocular pressure and optic nerve head characteristics in Cav-1 KO and WT mice. **(A)** Cav-1 KO mice show significantly higher IOP than WT. **(B–E)** Mean blood flow in optic nerve head and peripapillary vessels is lower in Cav-1 KO compared to WT mice. **(F)** Vessel density at the optic nerve head is not significantly different. **(G)** Colored fundus photographs do not show obvious differences in optic nerve head and peripapillary vessel morphology, **p < 0.01, ***p < 0.001.

We found that Cav-1 KO mice had modestly elevated IOP as compared to WT mice (11.40 ± 0.41 mmHg vs. 9.81 ± 0.17 mmHg), although this was statistically significant (*p* < 0.01, Figure 1A).

### Optic Nerve Head Imaging and Laser Speckle Flowgraphy

**FIGURE 2:**
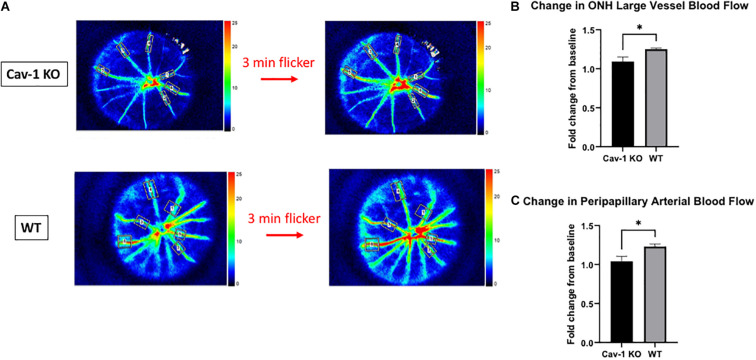
Blood flow changes after 3 min of light flicker stimulus indicates differences in the neurovascular coupling of response of Cav-1 KO compared to WT mice. **(A)** Representative LSFG images taken at the optic nerve head in Cav-1 KO and WT mice. **(B)** Cav-1 KO show a lesser increase in large vessel blood flow at the optic nerve head after flicker stimulus, compared to WT. **(C)** Cav-1 KO show a lesser increase in peripapillary arterial blood flow after flicker stimulus, compared to WT, *p < 0.05.

Colored optic nerve head photographs from Cav-1 KO mice demonstrated good optical clarity, with no gross differences in blood vessel morphology as compared to WT mice (Figure 1G). The mean blood flow in larger blood vessels (Cav-1 KO 14.76 ± 1.98 vs. WT 39.19 ± 3.94 AU, Figure 1B) as well as microvascular tissues (Cav-1 KO 6.38 ± 0.95 vs. WT 12.9 ± 0.61 AU, Figure 1C) at the optic nerve head, and both peripapillary arteries (Cav-1 KO 5.14 ± 0.36 vs. WT 10.02 ± 0.90 AU, Figure 1D) and veins (Cav-1 KO 6.51 ± 0.55 vs. WT 13.92 ± 1.50 AU, Figure 1E) were significantly lower in Cav-1 KO than WT mice (all *p* < 0.001) at baseline. Vessel density at the optic nerve head was not different in Cav-1 KO as compared to WT mice (Cav-1 KO 26.0 ± 0.81 vs. WT 26.44 ± 1.55%, Figure 1F), however. After exposure to 3 min of flicker stimulus, the fold change in blood flow from baseline was significantly lower in the large blood vessels of the optic nerve head and peripapillary arteries of Cav-1 KO as compared to WT mice (1.09 ± 0.06 vs. 1.25 ± 0.01 and 1.04 ± 0.06 vs. 1.23 ± 0.03, respectively, both *p* < 0.05, Figures 2A–C). There was no significant difference in the blood flow in the optic nerve head microvasculature or peripapillary veins of Cav-1 KO and WT mice, comparing baseline with 3 min of exposure to light flicker stimulus.

### Blood Vessel Morphology

**FIGURE 3:**
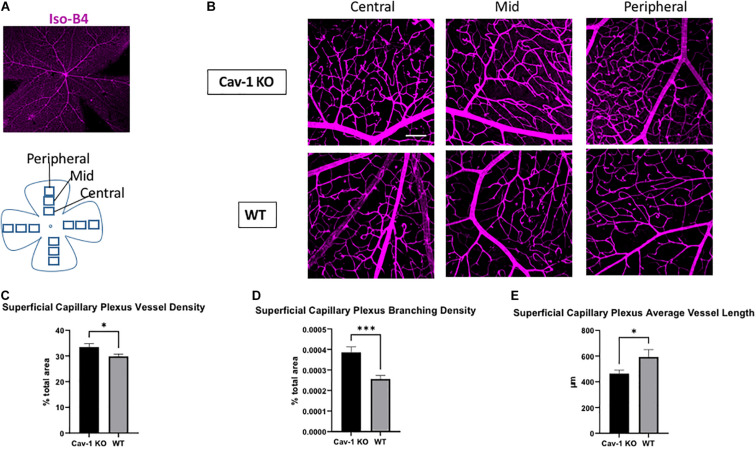
Analysis of superficial capillary plexus morphology. **(A)** Representative image of flat-mounted retina stained with endothelial cell marker Isolectin-B4 (Iso-B4, magenta). **(B)** Iso-B4 staining at various eccentricities from the optic nerve head in flatmounted retina, scale bar in white indicates 100 μm. **(C,D)** Cav-1 KO mice show increased superficial capillary plexus vessel density and branching density, compared to WT. **(E)** The average vessel length is the superficial capillary plexus is shorter in Cav-1 KO compared to WT, *p < 0.05, ***p < 0.001.

**FIGURE 4:**
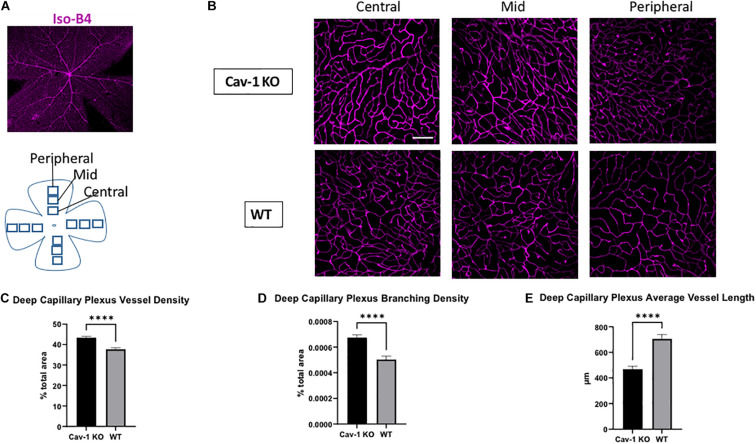
Analysis of deep capillary plexus morphology. **(A)** Representative image of flat-mounted retina stained with endothelial cell marker Isolectin-B4 (Iso-B4, magenta). **(B)** Iso-B4 staining at various eccentricities from the optic nerve head in flatmounted retina, scale bar in white indicates 100 μm. **(C,D)** Cav-1 KO mice show increased vessel density and branching density in the deep capillary plexus, compared to WT. **(E)** The average vessel length in the deep capillary plexus is shorter in Cav-1 KO compared to WT, ****p < 0.0001.

We found that the mean blood vessel density of the superficial capillary plexus was higher in Cav-1 KO compared to WT mice (Cav-1 KO 33.49 ± 1.30 vs. WT 29.85 ± 0.90%, *p* < 0.05, Figures 3A–C). The branching density of the superficial capillary plexus was also higher in Cav-1 KO compared to WT mice (Cav-1 KO 0.0003857 ± 2.64e-005 vs. WT 0.0003744 ± 1.65e-005%, *p* < 0.001, Figure 3D). Similar trends were noted in the deep capillary plexus (mean blood vessel density Cav-1 KO 43.35 ± 0.63 vs. WT 37.67 ± 0.73%, Figures 4A–C; mean branching density Cav-1 KO 0.0006741 ± 2.09e-005 vs. WT 0.0005033 ± 2.54e-005%, Figure 4D both *p* < 0.0001). However, the average vessel length was significantly shorter in the Cav-1 KO than WT mice, in both cases (superficial 463.4 ± 27.9 vs. 592.8 ± 57.6 μm, *p* < 0.05, Figure 3E; deep 469.0 ± 23.2 vs. 705.9 ± 34.48 μm, *p* < 0.0001, Figure 4E).

On comparing region-specific vessel characteristics in the SCP and DCP at different eccentricities from the optic nerve head, there was an identical trend to the global analysis. However, only the DCP showed a statistically significant difference in vessel density, branching density and average vessel length when comparing Cav-1 KO to WT mice. Comparisons of the SCP showed a statistically significant difference in branching density at the mid-region of the retina, while all other comparisons did not reach statistical significance (Supplementary Tables 1, 2).

### Retinal Ganglion Cell Function and Density

**FIGURE 5:**
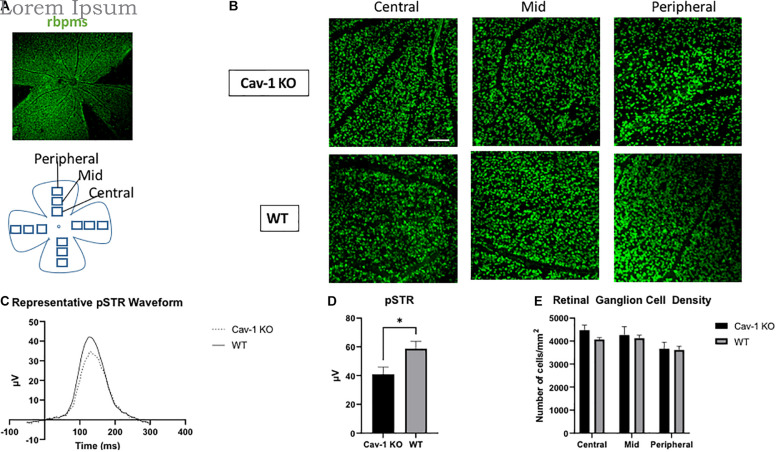
Retinal ganglion cell function and density. **(A)** Representative image of flat-mounted retina stained with retinal ganglion cell marker rbpms (green). **(B)** Rbpms staining at various eccentricities from the optic nerve head in flatmounted retina, scale bar in white indicates 100 μm. **(C)** Representative pSTR waveform in Cav-1 KO and WT mice. **(D)** Cav-1 KO mice show reduced pSTR compared to WT. **(E)** Mean retinal ganglion cell density is not significantly different in Cav-KO and WT mice, *p < 0.05.

Mean peak pSTR amplitude was lower in Cav-1 KO than WT mice (40.93 ± 5.02 vs. 58.70 ± 5.19 μV, *p* < 0.05, Figures 5C,D). RGC density in flatmounted retinas showed decreasing trends with increasing distance from optic nerve head as expected (Cav-1 KO central = 4476 ± 216.2, mid = 4265 ± 364.0, peripheral = 3665 ± 288.9/mm^2^; WT central = 4063 ± 85.7, mid = 3956 ± 105.5, peripheral = 3618 ± 173.0/mm^2^, Figures 5A,B,E) although there was no significant difference between Cav-1 KO and WT mice. A comparison of the global mean RGC density in Cav-1 KO (3919.32 ± 85.69 cells/mm2) and WT (4226.05 ± 172.99 cells/mm2) mice, also did not show any significant difference between both groups of animals (*p* = 0.09).

## Discussion

Overall, our findings suggest that the loss of Cav-1 leads to defective NVC at the optic nerve head, with associated changes in retinal vessel morphology and decreased electrophysiological function of RGCs. However, there was no significant difference in RGC density between Cav-1 KO and wild type mice.

Based on our current knowledge, there is little evidence regarding the role of Cav-1 in neurovascular coupling in the eye although Cav-1 has recently been described to play an important role in maintaining the physiological NVC response in the brain (Chow et al., 2020). Cav-1 is widely expressed among the cell-types that contribute to the neurovascular unit in the eye, suggesting that it may also be involved in neuro-retinal NVC (Li et al., 2012). Our study is the first to establish this relationship between Cav-1 and NVC in the eye. We also demonstrated resultant changes in vessel morphology such as higher vessel density, junction density and decreased average vessel length in flatmount retina images. Studies have suggested that macula vessel dropout is present even in the early stages of glaucoma (Suh et al., 2016). Optical coherence tomography angiography (OCT-A) of glaucoma patients also revealed decreased vessel density and narrowing of vessel caliber that results in impaired microcirculation at the ONH (Yip et al., 2019). Such vascular alterations accompany RGC losses, even though the temporal association remains unclear (Liu et al., 2015). In our study, we found that Cav-1 KO mice showed an increase in the density of retinal microvasculature and shortening of the average vessel length in both the superficial and deep capillary plexuses. This may be attributed to the role of Cav-1 in mediating angiogenesis, since Cav-1 has been described to downregulate vascular endothelial growth factor. Thus, it is possible that the depletion of Cav-1 conversely promotes angiogenesis and vascular endothelial cell proliferation (Liu et al., 1999). The effect of vascular branching patterns on tissue perfusion and oxygenation is largely unknown at present, although a recent study suggests that excessive vessel branching is associated with poorer blood flow in mouse retina (Mirzapour-Shafiyi et al., 2021). This is supported by our findings that the mean blood flow at the optic nerve head and peripapillary arteries and veins appeared to be decreased in Cav-1 KO as compared to WT mice at baseline. Therefore, it is possible that the altered vessel morphology observed in Cav-1 KO mice results in poorer neuro-retinal tissue perfusion leading to decreased RGC function.

Our study failed to show any structural RGC loss despite changes in vessel morphology. Nonetheless, we did find a decrease in electrical activity of RGCs in Cav-1 KO mice, which is suggestive of RGC dysfunction consistent with previous reports (Abbasi et al., 2020). Several models of experimental glaucoma have revealed that RGC dysfunction occurs early and precedes losses in optic nerve tissue (Porciatti, 2015). Hence, it appears that early effects of RGC injury are present with the depletion of Cav-1, even though structural losses are not yet observed.

Our present study lends support to the vascular theory of glaucoma pathogenesis by demonstrating defective vascular autoregulation accompanying RGC dysfunction and further identifies the loss of Cav-1 as a possible causative factor. Current treatment for glaucoma relies heavily on ocular hypotensive medications, however, these drugs are associated with significant side effects and a decrease in IOP does not always stop the progression of glaucoma (Porciatti, 2015). Our study has identified possible functional deficits in NVC in transgenic mice that exhibit deletion of Cav-1, prior to established structural changes affecting RGCs. This suggests that NVC impairment may be an earlier indicator of glaucomatous damage than structural measurements of RGC losses that are presently used in glaucoma clinics. Our findings also suggest the possibility of a therapeutic window for intervention to restore NVC, prior to apoptosis or irreversible structural losses taking place. Given the established role of Cav-1 in NVC, there is certainly potential for new therapeutic strategies that increase Cav-1 expression or augment its downstream signaling. For example, gene therapy to enhance Cav-1 expression in cortical neurons has been proposed to alleviate other forms of neurodegenerative disease (Leske et al., 2007), it is thus possible that similar approaches may have a resultant neuroprotective effect in the eye.

Our study can be viewed in light of certain limitations. Firstly, as our experiments were conducted solely in mice, we were unable to determine if our findings of impaired NVC were more prominent in human subjects with genetic mutations in Cav-1 as compared to those without. Secondly, while it is possible that the altered vessel morphology observed in Cav-1 KO mice is associated with reduced perfusion and hence oxygenation to RGCs, this was not demonstrated in our study. We were also unable to reliably measure blood pressure in these experiments, although other groups have determined that Cav-1 deficient mice do not show differences in mean systolic blood pressure as compared to WT (Desjardins et al., 2008). Finally, we have not proven the temporal sequence between vascular alterations and RGC injury, although it appears that the loss of Cav-1 contributes to both pathologies. It is possible that the modest IOP elevation seen in the Cav-1 KO compared to wild-type mice may only be sufficient to induce functional damage instead of structural losses. Outer retinal ERG responses were not measured in our study, although it has been reported that Cav-1 deletion is associated with decreased a- and b-wave amplitudes (Li et al., 2012). It is therefore possible that the reduction in pSTR noted in our Cav-1 KO mice was synchronous with, or downstream to outer retinal dysfunction. Others have reported methods to ascertain contributions from specific retinal cell-types that give rise to inner retinal ERG responses, using gain characteristics from successive components of recordings from control animals although this was not performed in our study (Nguyen et al., 2013). Further work is necessary to determine if NVC deficits and functional impairment of RGCs related to Cav-1 depletion can occur in the absence of raised IOP. We also found that the deep capillary plexus was altered in Cav-1 KO, which may be in keeping with previous reports of diminished outer retinal electrophysiological signaling (Abbasi et al., 2020). However, it was not possible to examine the NVC in the outer retinal layers or peripheral retina using our LSFG set-up. Future work using plexus-resolved optical coherence tomograph angiography may enable studies of NVC in the deep capillary plexus as well.

Cav-1 deletion has previously been described to be associated with altered endothelial-mural cell interactions in retinal blood vessels, and breakdown of the blood-retinal barrier (BRB) (Gu et al., 2014a). The interactions between BRB permeability and neurovascular coupling in the eye have not been described yet, to the best of our knowledge. It is evident from studies in the brain, however, that blood-brain barrier permeability is modulated by neuronal function and vice versa (Kaplan et al., 2020). Similar mechanisms may be at play in the retina as well. Further research into the interactions between BRB and caveolin-1 may provide valuable insight into specific factors that influence neurovascular coupling in glaucoma.

In conclusion, Cav-1 plays a role in regulating neurovascular coupling in the eye. Here, we show that the loss of Cav-1 may predispose to vascular dysfunction and decreased RGC signaling in the absence of structural loss. These findings indicate possible new avenues for the earlier detection of glaucoma and novel neuroprotective treatments for this important blinding disease.

## Data Availability Statement

The raw data supporting the conclusions of this article will be made available by the authors, without undue reservation.

## Ethics Statement

The animal study was reviewed and approved by the SingHealth Institutional Animal Care and Use Committee.

## Author Contributions

RC, BT, and LS contributed to conception and design of the study. YL, VY, JL, and CW performed the data collection. RC performed the statistical analysis. JL wrote the first draft of the manuscript. YL, CW, and RC wrote sections of the manuscript. All authors contributed to manuscript revision, read, and approved the submitted version.

## Conflict of Interest

The authors declare that the research was conducted in the absence of any commercial or financial relationships that could be construed as a potential conflict of interest.

## Publisher’s Note

All claims expressed in this article are solely those of the authors and do not necessarily represent those of their affiliated organizations, or those of the publisher, the editors and the reviewers. Any product that may be evaluated in this article, or claim that may be made by its manufacturer, is not guaranteed or endorsed by the publisher.

## Supplementary Material

Supplementary Table 1 Superficial capillary plexus characteristics at increasing distances from the optic nerve head. Cav-1 KO mice showed a trend toward increased superficial capillary plexus vessel density and branching density in all regions compared to WT, however, only branching density in the mid-region showed a statistically significant difference. The average vessel length in the superficial capillary plexus was shorter in Cav-1 KO in all regions compared to WT, although this did not reach statistical significance, ^∗∗^*p* < 0.01.

Supplementary Table 2 Deep capillary plexus characteristics at increasing distances from the optic nerve head. Cav-1 mice showed increased deep capillary plexus vessel density and branching density, with decreased average vessel length as compared to WT mice in all regions of the retina, ^∗^*p* < 0.05, ^∗∗∗^*p* < 0.001.
