# Supplementary material for: Loss of Caveolin-1 Impairs Light Flicker-Induced Neurovascular Coupling at the Optic Nerve Head
Source: Front Neurosci. 2021 Nov 8;15:764898. doi: 10.3389/fnins.2021.764898 (PMC8606647; doi:10.3389/fnins.2021.764898)
Supplement: Supplementary Table 1 — Superficial capillary plexus characteristics at increasing distances from the optic nerve head. Cav-1 KO mice showed a trend toward increased superficial capillary plexus vessel density and branching density in all regions compared to WT, however, only branching density in the mid-region showed a statistically significant difference. The average vessel length in the superficial capillary plexus was shorter in Cav-1 KO in all regions compared to WT, although this did not reach statistical significance, ∗∗p < 0.01. [file Table_1.docx]

Supplemental Table 1.

| **SCP** | **Vessel Density (Mean±SEM %)** | |  |
| --- | --- | --- | --- |
|  | Central | Mid | Peripheral |
| Cav-1 KO | 34.48±1.83 | 32.32±3.42 | 33.72±2.78 |
| WT | 31.39±1.39 | 28.33±1.69 | 29.8±1.50 |
| p-value | 0.47 | 0.25 | 0.24 |
|  |  |  |  |
|  | **Branching Density (Mean±SEM %)** | |  |
| Cav-1 KO | 0.00039±4.00E-05 | 0.00041±7.56E-05 | 0.00035±4.81E-05 |
| WT | 0.0003±2.48E-05 | 0.00023±2.74E-05 | 0.00025±2.17E-05 |
| p-value | 0.16 | 0.01** | 0.11 |
|  |  |  |  |
|  | **Vessel Length (Mean±SEM μm)** | |  |
| Cav-1 KO | 496.53±55.39 | 462.06±77.16 | 426.22±53.25 |
| WT | 614.58±89.04 | 530.56±29.36 | 590.72±174.15 |
| p-value | 0.22 | 0.44 | 0.34 |
