# Supplementary material for: Loss of Caveolin-1 Impairs Light Flicker-Induced Neurovascular Coupling at the Optic Nerve Head
Source: Front Neurosci. 2021 Nov 8;15:764898. doi: 10.3389/fnins.2021.764898 (PMC8606647; doi:10.3389/fnins.2021.764898)
Supplement: Supplementary Table 2 — Deep capillary plexus characteristics at increasing distances from the optic nerve head. Cav-1 mice showed increased deep capillary plexus vessel density and branching density, with decreased average vessel length as compared to WT mice in all regions of the retina, ∗p < 0.05, ∗∗∗p < 0.001. [file Table_2.docx]

Supplemental Table 2.

| **DCP** | **Vessel Density (Mean±SEM %)** | |  |
| --- | --- | --- | --- |
|  | Central | Mid | Peripheral |
| Cav-1 KO | 45.16±1.06 | 43.32±1.06 | 41.58±1.06 |
| WT | 40.11±4.03 | 36.81±3.83 | 36.08±3.58 |
| p-value | 0.0026*** | 0.00079*** | 0.0018*** |
|  |  |  |  |
|  | **Branching Density (Mean±SEM %)** | |  |
| Cav-1 KO | 0.00071±2.75E-05 | 0.00067±3.95E-05 | 0.0006±4.33E-05 |
| WT | 0.00054±6.02E-05 | 0.00049±6.34E-05 | 0.00049±6.02E-05 |
| p-value | 0.00083*** | 0.0070*** | 0.023* |
|  |  |  |  |
|  | **Vessel Length (Mean±SEM μm)** | |  |
| Cav-1 KO | 497.12±42.95 | 482.30±47.81 | 426.69±30.22 |
| WT | 798.64±90.32 | 670.04±91.56 | 649.13±80.30 |
| p-value | 0.00016*** | 0.024* | 0.0012*** |
